# Taurine alleviates hyperuricemia-induced nephropathy in rats: insights from microbiome and metabolomics

**DOI:** 10.3389/fnut.2025.1587198

**Published:** 2025-06-18

**Authors:** Xiujuan Yang, Hengxi Li, Daermu Qumu, Binhui Han, Mukaram Amatjan, Qiyao Wu, Lanting Wei, Bo Li, Mengxue Ma, Junjie He, San Wang, Yingzhi Yu, Xiaoni Shao

**Affiliations:** ^1^College of Pharmacy and Food, Southwest Minzu University, Chengdu, China; ^2^Xinjiang Institute of Materia Medica, Ürümqi, China

**Keywords:** taurine, hyperuricemia, nephropathy, gut microbiota, metabolomics

## Abstract

**Background:**

Gut microbiota play a critical role in developing hyperuricemic nephropathy (HN). We previously found that sulfur-containing amino acid taurine (T) has nephroprotective effects in hyperuricemia (HUA) rats. However, the mechanism is still unclear. To investigate the underlying mechanism of T, rats were fed adenine and ethambutol hydrochloride for the introduction of HN.

**Methods:**

Pathological changes in the kidney were assessed using hematoxylin and eosin staining. 16S rRNA sequencing and metabolomics analyzed changes in the gut microbiota and fecal metabolism, and *in vitro* experiments were conducted to investigate the potential action and mechanism of T against HN.

**Results:**

*In vitro* results demonstrated that T could inhibit NF-κB, IL-1β, IL-6, TNF-α, and ROS in UA-induced HK-2 cells. It also improved renal function, ameliorated renal fibrosis, and reversed enteric dysbacteriosis in HN rats. These results showed that T protects against HN through the modulation of metabolites mediated by the gut microbiota. Meanwhile, gut microbiota included *Lactobacillus* and *Lachnospiraceae NK4A136 group* showed correlations with nephroprotective profiles of T. The combined analysis of 16S rRNA gene sequencing and untargeted metabolomics indicated that the anti-HN effects of T could be achieved through phenylalanine metabolism, caffeine metabolism, nicotinate and nicotinamide metabolism, retinol metabolism, and tryptophan metabolism.

**Conclusion:**

These findings suggest that the potential protective mechanism of T for HN is not only related to altered metabolic pathways and downregulation of inflammatory cytokines but also to the reciprocal regulation of microbiota structure and metabolism.

## 1 Introduction

Hyperuricemia (HUA) has become one of the most frequent metabolic disorders in the world, with a dramatic increase in incidence globally (1). HUA results from increased uric acid (UA) production or insufficient renal excretion. UA is the ending product of purine or nucleotide metabolism. One-third of UA is excreted through the intestine and two-third through glomerular filtration (2). The kidney injury caused by HUA is known as hyperuricemia-induced nephropathy (HN) and is characterized by atherosclerosis, glomerular hypertension, urate deposition, and tubulointerstitial fibrosis, culminating in the development of end-stage kidney disease (3). UA can cause precipitation and obstruction in the renal tubules, which can lead to acute kidney injury, especially in the case of tumor lysis syndrome (4). UA may also cause endothelial dysfunction, activation of the renin–angiotensin–aldosterone system (RAAS), inflammation, and oxidative stress, leading to chronic kidney disease (CKD) and its progression (5).

Currently, the main classes of drugs that are available for the treatment of HN include xanthine oxidase (XOD) inhibitors, UA excretory drugs, and recombinant uricase supplements. However, these drugs have clinical side effects such as hepatotoxicity and nephrotoxicity (6). There have been no studies related to the treatment of HN from the perspective of food therapy. As a result, the search for food additives or dietary therapeutic agents with fewer adverse effects and greater therapeutic action as substitutes for current HN-treating drugs is crucial.

Taurine (T) is a sulfur-containing amino acid that is abundant in mammalian cells (7). It plays an important role in the growth and development of brain cells and is a non-proteinogenic essential amino acid (8, 9). T has antioxidant, anti-inflammatory, and neuromodulatory pharmacological effects (10, 11). A study showed that T was effective in reducing UA, blood urea nitrogen (UREA), and creatinine (CREA) levels in alloxan-induced diabetic rats and also reduced serum UA levels in streptozotocin-induced diabetic rats (12, 13). T reduces elevated blood glucose and pro-inflammatory cytokine levels, reduces kidney oxidative stress, a process mediated mainly by reducing XOD activity, advanced glycation end product (AGE) formation, and inhibition of the p47 phox/cytochrome P450 family 2, subfamily D, polypeptide 1 (CYP2D1) pathway, family 2, subfamily E, polypeptide 1 (CYP2E1) pathway. It also improves renal function and protects renal tissue from alloxan-induced apoptosis by modulating B-cell lymphoma-2 (Bcl-2) family and caspase-9/3 proteins (12). Zhu et al. (14) demonstrated that the number of *Bifidobacteria* and *Lactobacilli* in the gut changed significantly after T treatment. Shen et al. (15) have demonstrated that supplementation with 0.8% T can significantly enhance the synthesis of carbohydrates, the digestion and absorption of proteins, and the deposition of fat, thus promoting growth and development. However, the mechanism of T action in HN remains unclear.

In recent years, the relationship between microbiota and its host has garnered significant attention, with the gut–renal axis theory effectively illustrating the two-way communication between gut microbiota and kidney disease. Impaired renal function may lead to dysbiosis of the gut flora. Meanwhile, changes in the gut flora enable endotoxins and bacterial metabolites to accelerate renal injury by disrupting the gut mucosal barrier and inducing systemic inflammation transferred from the gut to the systemic circulation (16). Gut flora analyses showed that in the HN model group, conditional pathogens such as *Flavobacterium*, *Myroides*, *Corynebacterium*, and *Alcaligenaceae* increased significantly, while bacteria producing short-chain fatty acids such as *Blautia* and *Roseburia* decreased significantly (17). T is involved in multiple physiological processes, including bile acid conjugation (18, 19). Miao et al. (20) demonstrated that T can regulate the immune response after *S. uberis* infection and thus prevent mammary tissue damage. In addition, Zhu et al. (14) indicated that the number of *bifidobacteria* and *lactobacilli* in the gut changed significantly after T treatment.

Metabolomics is one of the rapidly growing fields in the life sciences that uses sophisticated analytical chemistry techniques and reliable statistical methods to characterize endogenous metabolites and their patterns of change in biological systems (21). It has been successfully applied to biomarker discovery, disease pathology, and drug toxicity prediction (22, 23). Metabolic analysis of small molecules produced by the gut microbiota in host feces or tissues provides greater insight into metabolite changes and the physical health of the host. Recent studies have shown that HUA is strongly associated with the development and severity of the metabolic syndrome (24). Metabolomics is part of systems biology. A study exploring the effects of T on dyslipidemia in a high-fat diet-induced rat model using an NMR-based metabolomics method showed that T regulated creatine, methionine, glutamine, and threonine, as well as lipid metabolism (25). Shen et al. (15) found that supplementing tilapia with 0.8% T significantly increased their ability to synthesize carbs, digest and absorb protein, and store fat, thus promoting their growth and development.

In this study, we adopted a novel integrated approach to investigate the biological mechanisms of T therapy for HN based on gut flora and metabolites. To begin with, the flora and pathways were analyzed in accordance with the data of gut flora. Subsequently, metabolomics was employed to analyze the effects of T on metabolites in HN model rats. Eventually, the results of gut flora and metabolomics were combined and analyzed for the purpose of investigating the food therapy mechanisms of T. This study provides a scientific basis for the accurate screening of biomarkers and helps to establish the link between flora and biomarkers. Importantly, we found that T attenuated renal fibrosis in rats with HUA.

## 2 Materials and methods

### 2.1 Reagents

Adenine (A108804) was purchased from Shanghai Aladdin Biochemical Technology Co., Ltd. (Shanghai, China). Ethambutol hydrochloride tablets (H51020917) were supplied by Chengdu Jinhua Pharmaceutical Co., Ltd. (Chengdu, China). T (T818825) (high-performance liquid chromatography (HPLC) purity 99%) was provided by Macklin Reagent (Shanghai, China). SYBR Green (1725120) was purchased from Bio-Rad Laboratories (Hercules, California, USA). RNase-free H_2_O (BL510B) and 4% paraformaldehyde (BL539A) were purchased from Biosharp Life Sciences (Beijing, China). Ethanol (100092683), xylene (10023418), and neutral gum (10004160) were bought from Sinopharm Chemical Reagent Co. Ltd. (Shanghai, China). Hematoxylin and eosin (H&E) dye solution set (G1003), environmentally friendly dewaxing solution (G1128), Masson’s trichrome dye solution set (G1006), hematoxylin differentiate solution (G1039), and periodic acid Schiff (PAS) dye solution set (G1008) were provided by Wuhan Servicebio technology CO., LTD. (Wuhan, China). Pancreatic enzyme cell digestive fluid (BL501A), Dulbecco’s modified Eagle medium (DMEM) (8123470), and phosphate buffer saline (PBS) (BL302A) were supplied by Beijing Lange Technology Co., Ltd. (Beijing, China). Methanol (A456-4), formic acid (A117-50), and ammonium acetate (A114-50) are purchased from Thermo Fisher Scientific (Waltham, MA, USA).

### 2.2 Animals and treatment

**Figure 1:**
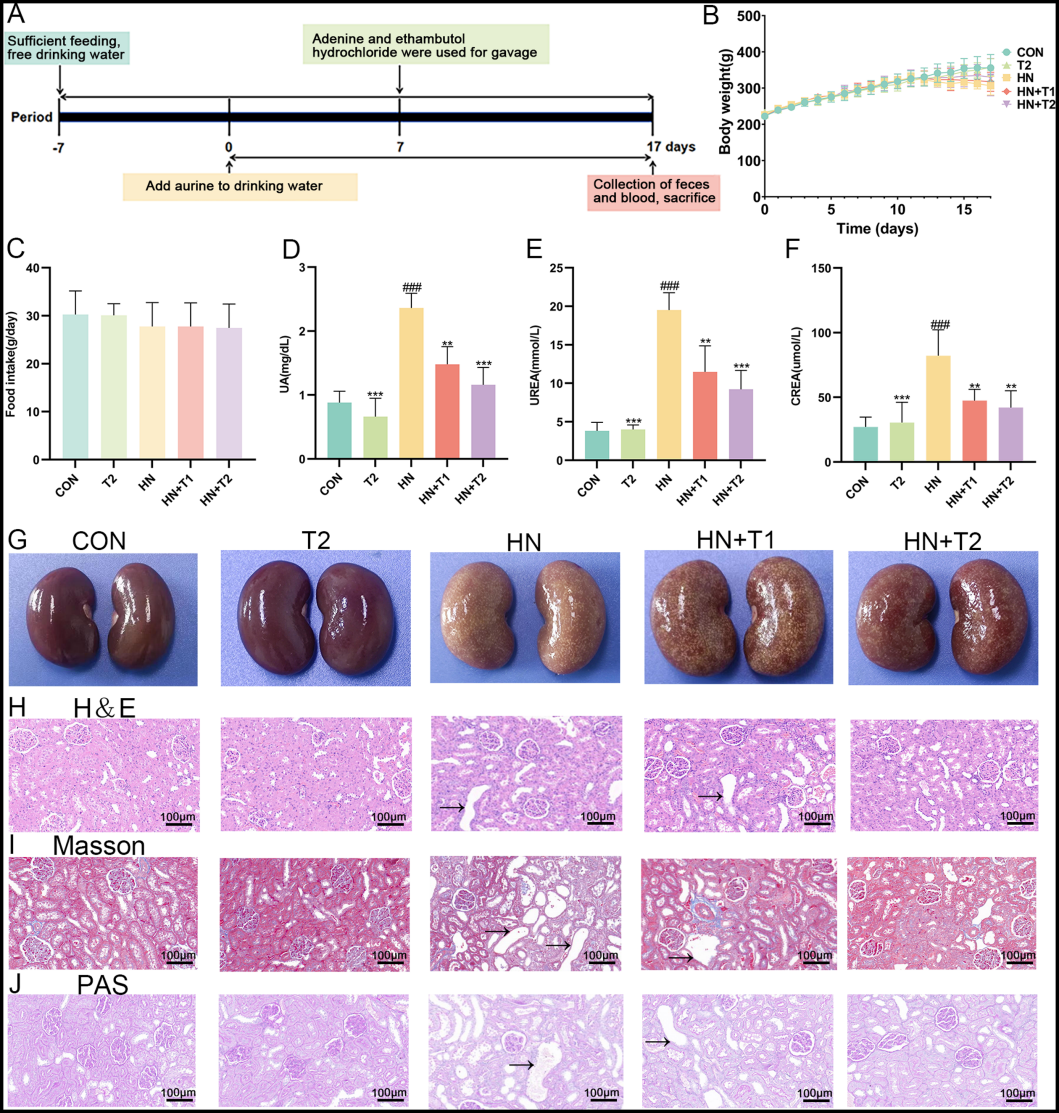
Effects of T on the serum UA level and renal function in HN rats. **(A)** Study design of the present study. **(B)** Body weight. **(C)** Food intake. **(D)** Serum UA. **(E)** Serum UREA. **(F)** Serum CREA. **(G)** Photographs of kidney tissues from different groups. **(H)** Histopathological H&E staining analysis of kidney tissue. **(I)** Histopathological Masson staining analysis of kidney tissue. **(J)** Histopathological PAS staining analysis of kidney tissue. Values were expressed in mean ± SEM (*n* = 6/group). ^#^*p* < 0.05, ^##^*p* < 0.01, ^###^*p* < 0.001 vs. CON, ^*^p < 0.05, ^**^p < 0.01, ^***^p < 0.001, vs. HN.

All animal experiments were carried out in line with the standards of the Chinese Society of Laboratory Animals and with the approval of the Animal Care and Use Ethics Committee of Southwest Minzu University (SMU - 20239008). Healthy Wistar rats (male, 6–8 weeks, SPF, 180–200 g) were obtained from Chengdu Dossy Experimental Animals Co., Ltd. (Chengdu, China). All rats were acclimated for 1 week before the experiments (humidity 55 ± 5%, temperature 23 ± 1°C, and 12-h/12-h light/dark cycle) and were fed and watered ad libitum. A total number of 30 rats were randomly and equally divided into five groups (6 rats per group): control group (CON), HN model group (HN), taurine control group (T2), low-dose T treatment group (HN + T1), and high-dose T treatment group (HN + T2). All rats of the HN, HN + T1, and HN + T2 groups were intragastrically administered a mixture of adenine (100 mg/kg) and ethambutol hydrochloride (250 mg/kg) every day for consecutive 7 days to induce HN. Rats given 1% T in the drinking water were designated as the HN + T1 group and those given 2% T as the HN + T2 group. The rats in the T2 group were only administered 2% T in the drinking water during the entire experimental period. For rats in the T2, HN + T1, and HN + T2 groups, T was added to the drinking water for 10 days prior to treatment with adenine and ethambutol hydrochloride. For the duration of the experiment, the rats in the CON group received only drinking water. The same chow was fed to all rats. During the study, no animals died. On day 17, rats were individually placed in metabolic cages for 24 h, and fecal samples were collected for 24 h after the last intragastric administration. Then, the rats were euthanized, and blood, fecal, and renal samples were collected for further analysis (Figure 1A).

### 2.3 Measurement of biochemical indexes

Blood samples were centrifuged in a Centrifuge (Cence H2050R, Hunan, China) at 4°C and 3,000 rpm for 15 min to obtain serum. The evaluation of UA, CREA, and UREA was measured by an Automatic Biochemistry analyzer (Cobas C311, Roche, Switzerland).

### 2.4 Histological examination

Renal tissues were fixed with 4% paraformaldehyde buffer and then dehydrated with gradient ethanol to make them transparent, embedded in paraffin, and sectioned into sections 4 μm thick. The kidney sections were stained with H&E, PAS, or Masson’s trichrome (Masson). Sections were observed, and images were acquired for analysis with an upright light microscope (Eclipse E100, Nikon Instruments, Japan), and ultimately, each set of slices was meticulously analyzed.

### 2.5 Cell culture and cell counting Kit-8 (CCK-8) assay

Human renal proximal tubule cells (HK-2, ATCC CRL-2190, Qingqi Biotechnology Development Co., Ltd., Shanghai, China) were cultured in DMEM (Thermo Fisher Biochemical Products Co., Ltd., Beijing, China) supplemented with 5% fetal bovine serum (FBS, Viva Cell Biosciences, Shanghai, China), 0.5% penicillin, and streptomycin (37°C, 5% CO_2_). HK-2 cells were cultured in 96-well plates and then added with increasing concentrations of UA (5, 10, 20, 40, 80, and 160 mg/dL) and T (5, 10, 20, 40, 80, and 160 mM) for 24, 36, or 48 h, respectively. Then, 10 μL of Cell Counting Kit (CCK-8, Selleck Chemicals, Houston, TX, USA) was added to each well and then incubated at 37°C for another 1 h. Then, the absorbance of each sample was measured at 450 nm. The cell viability was determined by the CCK-8 method according to the protocol provided by the manufacturer.

### 2.6 Quantitative real-time polymerase chain reaction (qRT-PCR)

The kidney tissues of each group of rats were taken into a pre-cooled mortar, liquid nitrogen was added and ground into powder, the total RNA was extracted, and cDNA was synthesized. Total RNA from the kidney samples of rats and HK-2 cells was extracted using the AxyPrep™ Multisource Total RNA Maxiprep Kit (Corning, Suzhou, China) according to the manufacturer’s instructions and then reverse-transcribed with the PrimeScript™ RT reagent Kit with gDNA Eraser (Takara Bio, Beijing, China). Quantitative PCR experiments were carried out using iTaq™ Universal SYBR Green Supermix Dye in a reaction volume of 20 μL. The cycling programs are established as below: the initial cycle of 95°C for 10 min, followed by 40 cycles of 95°C for 10 s and 60°C for 30 s. Normalization was performed using the reference gene GAPDH and analyzed using the 2^−∆∆CT^ method. The sequences of the qRT-PCR primers are listed in Table 1.

**Table 1:** Primer sequences for real-time PCR of target genes used in the study.

Primer name		Primer sequence (5′ to3′)		bp
		Forward primer	Reverse primer	
Rat	URAT1	ATGTGAGGATGGCTGGGTTTAC	CCAGACACAGACACCAGAAGATA	216
	ABCG2	TTGGACTCAAGCACAGCAAATG	TGAGTTTCCCAGAAGCCAGTAAG	148
	OAT1	CCGGATCAATGGGAAACAAGAAG	AGCATGGAGAGACAGAGGAAGAG	159
	NF-κB	CTGTGTGACAAAGTGCAGAAAGA	GATCGTCTGTGTCTGGCAAGTA	241
	IL-6	ATACCACCCACAACAGACCAGTA	AGTGCATCATCGCTGTTCATACA	136
	IL-1β	AAGGAGAGACAAGCAACGACAAA	CTGCTTGAGAGGTGCTGATGTA	240
	GLUT9	TGCATTGGCGTGTTTTCTGG	GTTTGGAAGGCTTTCGTGGC	197
	TNF-α	ACGATGCTCAGAAACACACG	TCCACTCAGGCATCGACATT	131
	GAPDH	GAAGGTCGGTGTGAACGGAT	CCCATTTGATGTTAGCGGGAT	250
Human	URAT1	GACACCATCCAAGATGTGCAGAA	TGGAAATGGAGGTCTTTGCTTTC	182
	ABCG2	CTCTTCGGCTTGCAACAACTATG	CTCCAGACACACCACGGATAAA	132
	OAT1	GCACCTTGATTGGCTATGTCTAC	CTTCTTCCCGCTTCCCATTGAT	234
	NF-κB	GAGGATGGGATCTGCACTGTAAC	GTGCACCAAGAGTCCAGGATTAT	162
	IL-6	TCTCTGGGAAATCGTGGAAATGA	ACTCCAGAAGACCAGAGGAAATT	163
	IL-1β	AGTGGCAATGAGGATGACTTGTT	AAAGAAGGTGCTCAGGTCATTCT	237
	GLUT9	ACTGCCATCTTTATCTGCATTGG	CTCTTGGGAAACGTCTGCTTTAC	243
	TNF-α	GCTGCACTTTGGAGTGATCG	TCACTCGGGGTTCGAGAAGA	108
	GAPDH	GGAGTCCACTGGCGTCTTCA	GTCATGAGTCCTTCCACGATACC	240

### 2.7 ROS detection

The HK-2 cells were planted and treated as described in the previous section. The 2'7'-dichlorodihydrofluorescein diacetate (DCFH-DA) was used to determine ROS levels according to the manufacturer’s instructions. ROS green fluorescence was captured using fluorescence microscopy. Relative fluorescence intensity was calculated by Image-Pro Plus (Media Cybernetics, FL, USA).

### 2.8 Microbial 16S rRNA gene sequence and metagenomic analyses

Fresh fecal samples were collected from five groups of rats (*n* = 6 rats in each group) for 16S rRNA gene sequencing. Genomic DNA was extracted from the samples using CTAB or SDS and checked for purity and concentration. The gDNA samples were purified using the ZymoBIOMICS DNA Microprep Kit (Zymo Research, California, USA), the integrity of the gDNA was checked using 0.8% agarose electrophoresis, and then, the concentration of nucleic acids was checked using the Multi-Wavelength Measurement System (Tecan Infinite F200, Switzerland). On the basis of the sequenced region, specific primers with index sequences were synthesized and used to amplify the V4 region of the sample. The V4 region was amplified with the primer 5′-3′: 515F (5’-GTGYCAGCMGCCGCGGTAA-3′) and 806R (5’-GGACTACHVGGGTWTCTAAT-3′) by a thermocycler PCR system (GeneAmp 9,700, ABI, USA), and the target fragments were electrophoresed on a 2% agarose gel. Samples that passed the assay were taken for recovery of the destination bands, which were recovered using the Zymoclean™ Gel DNA Recovery Kit (Zymo Research, CA, USA), quantified using a Qubit® 2.0 Fluorometer (Thermo Fisher Scientific, MA, USA), and finally mixed in equimolar amounts.

Sequences were processed for noise reduction and chimera removal using QIIME2 according to the Deblur algorithm to generate ASV feature tables and feature sequences. A species classification dataset was constructed for the SILVA database using a classifier based on the Naïve Bayes algorithm, and this dataset was used for species annotation of ASV feature sequences. Feature sequences were multiplexed using QIIME2, and evolutionary trees were constructed using its built-in FastTree plug-in. Homogenization was done for each sample, and resampling was done using the least amount of data in the sample as the criterion. Community analyses were performed using the R language for various data transformations and the ggplot2 software package for plotting graphs. The PD index was calculated using the Picante package, and the other indices were calculated using the Vegan package. The Wilcoxon order test was performed using the wilcox.test function of the statistical package, and the Kruskal–Wallis rank-sum test was performed for both groups using the Kruskal.test function. Multiple comparisons were performed with the agricolae package. β-diversity analysis was conducted using R. UniFrac distances were computed via the GuniFrac package, while Bray–Curtis and Jaccard distances were calculated with the vegdits function of the Vegan package. The principal coordinate analysis (PCoA) was carried out by means of the ape package. The principal component analysis (PCA) and non-metric multidimensional scaling (NMDS) analyses were conducted using the vegan package. Cluster analysis employed the hclust function of the stats package. ANOSIM and PERMANOVA were computed using the anosim function and adonis function of the vegan package, respectively. LefSe analysis was performed using the LEfse tool.^1^ Random forests were analyzed using the random Forest package in R. Metastases are analyzed using R scripts, and the computational steps can be found at https://journals.plos.org/ploscompbiol/articleid=10.1371/journal.pcbi.1000352. Community function was predicted using PICRUSt2. Community metabolic and ecological functions were predicted using FAPROTAX.

### 2.9 Ultraperformance liquid chromatography–tandem mass spectrometry (UPLC-MS/MS) method for metabolomics

UHPLC–MS/MS analyses were done with a Vanquish UHPLC system (Thermo Fisher Scientific, Germany) coupled with an Orbitrap Q Exactive™ HF or Q Exactive HF-X mass spectrometer (Thermo Fisher Scientific, Germany).

Fresh fecal samples were collected from five groups of rats for metabolomics analysis (6 rats per group). Approximately 100 mg of a fecal sample was put into an EP tube. Then, 500 μL of 80% methanol–water solution was added. After vortexing, it was allowed to stand in an ice bath for 5 min. Then, the mixture was centrifuged at 15000 g and 4°C for 20 min. A specific amount of the supernatant was diluted with mass spectrometry-grade water until the methanol content reaches 53%. The diluted solution was vortexed and centrifuged it again at 15000 g and 4°C for 20 min. Thereafter, the supernatant was collected for chromatographic analysis.

Samples were analyzed with a 2.1 mm × 100 mm, 1.9 μm Hypersil Gold column (Waters, Germany). In ESI positive mode, the mobile phase consisted of A = 0.1% formic acid and B = methanol. In ESI negative mode, the mobile phase was A = 5 mM ammonium acetate (pH = 9.0), B = methanol. The gradient was 2% B for 1.5 min, with a linear increase to 98% in 10.1 min and was retained for 1.9 min. The gradient flow rate was 0.2 mL/min, and the column temperature was maintained at 40°C. Then, 2 μL of each sample was injected. The ESI source is set up as follows: Q Exactive™ HF mass spectrometer was operated in positive/negative polarity mode with spray voltage of 3.5 kV, sheath gas flow rate of 35 psi, and aux gas flow rate of 10 L/min, capillary temperature of 320°C, S-lens RF level of 60, Aux gas heater temperature of 350°C, and MS/MS secondary scans are data-dependent scans.

Raw data files generated by UPLC-MS/MS were processed using Compound Discoverer 3.3 (CD3.3, Thermo Fisher Scientific) for peak alignment, peak selection, and metabolite quantification. The main parameters were set as follows: peak area corrected with the first QC, actual mass tolerance of 5 ppm, signal intensity tolerance of 30%, and minimum intensity. The molecular formula was then predicted based on the addition ions, molecular ion peaks, and fragment ions using data normalized from peak intensity to total spectral intensity. The peaks were then compared to the mzCloud,^2^ mzVault, and Mass List databases to provide accurate qualitative and relative quantitative results. Statistical analyses were performed using the statistical software R (R - version 3.4.3), Python (version 2.7.6), and CentOS (CentOS version 6.6). When the data were not normally distributed, standardize them using the formula: the sum of sample metabolite quantitation values divided by the sum of QC sample metabolite quantitation values to obtain relative peak areas. Compounds with relative peak area CVs greater than 30% were excluded from the QC samples to produce the final results for metabolite identification and relative quantification.

Ultimately, these metabolites were annotated using the KEGG database,^3^ HMD database,^4^ and LIPID Maps database.^5^

### 2.10 Statistical analysis

At least three independent experiments were conducted for each assay unless otherwise noted. The study data are expressed as mean ± standard error of the mean (SEM). Data were analyzed using one-way analysis of variance (ANOVA) followed by Tukey’s *post-hoc* test or independent Student’s *t*-test. The Spearman test analyzed the correlations among gut microbiota genera, metabolites, and serum biochemical indexes. GraphPad Prism 8.0.2 software (San Diego, CA, USA) was used for all statistical analyses and graphs. No exclusion criteria were involved in the analysis. Statistically significant differences were represented at a *p*-value of < 0.05.

## 3 Results

### 3.1 Taurine lowered UA and alleviated kidney injury in HN rats

As presented in Figures 1B,C, there were no notable changes in the body weight and food intake of the HN rats. Similarly, intervention with T did not induce noticeable alterations in body weight or food intake in rats. Significantly (*p* < 0.001) increased serum UA level was observed for the HN group (2.36 ± 0.23 mg/dL compared to 0.88 ± 0.18 mg/dL of the CON group), which corroborated the successful establishment of the HUA model. T resumed the serum UA level, accompanied by an improvement in renal dysfunction parameters. Importantly, T at 1 and 2% doses remarkably decreased the serum levels of UA, CREA, and UREA in HN rats. Notably, 2% T showed a significant nephroprotective effect, UA decreased to 1.16 ± 0.27 mg/dL, UREA decreased from 19.52 ± 2.25 mmol/L to 9.22 ± 2.46 mmol/L, and CREA decreased from 82.2 ± 19.98 μmol/L to 42.0 ± 13.04 μmol/L (Figures 1D−F, *p* < 0.05). The aforementioned results collectively suggested that T may confer protection against HN.

### 3.2 Taurine mitigated kidney fibrosis in HN rats

The histological study of kidney in various groups is presented in Figures 1G–J. The renal structure in the CON group had a distinct outline and seemed normal. There were no obvious anomalies in the medulla, the brush border was arranged regularly and neatly, and the epithelial cells were spherical and undamaged. There was no discernible inflammation or interstitial hyperplasia in the interstitial tissue, which served as the connective tissue between the urine tubules. On the other hand, the HN group’s renal cortex showed localized lymphocytic infiltration and a slight amount of tubular atrophy, as seen by the black arrows. Nevertheless, the renal histopathology’s inflammatory alterations were much reduced by T supplementation, suggesting that T had a strong protective impact against inflammatory changes brought on by HUA (Figure 1H). Given that HUA results in kidney fibrosis, which is defined by collagen deposition, the identification of collagen accumulation in the kidneys of HN rats was conducted through the utilization of Masson staining. As anticipated, the kidneys of HN rats exhibited multiple blue-stained areas within the tubulointerstitial compartment (Figure 1I). T demonstrated efficacy in attenuating renal interstitial fibrosis, accompanied by a reduction in collagen deposition. In PAS staining, the HN group shows thickening of the glomerular basement membrane, whereas the tubular basement membrane exhibits thickening accompanied by wrinkling and deformation. In comparison, the HN + T2 group demonstrated improved thickening of the glomerular basement membrane, while the thickness of the tubular basement membrane was normal (Figure 1J).

### 3.3 Taurine resumed the modified expression level of UA transporters and mitigated the inflammatory response in the kidneys of HN rats

**Figure 2:**
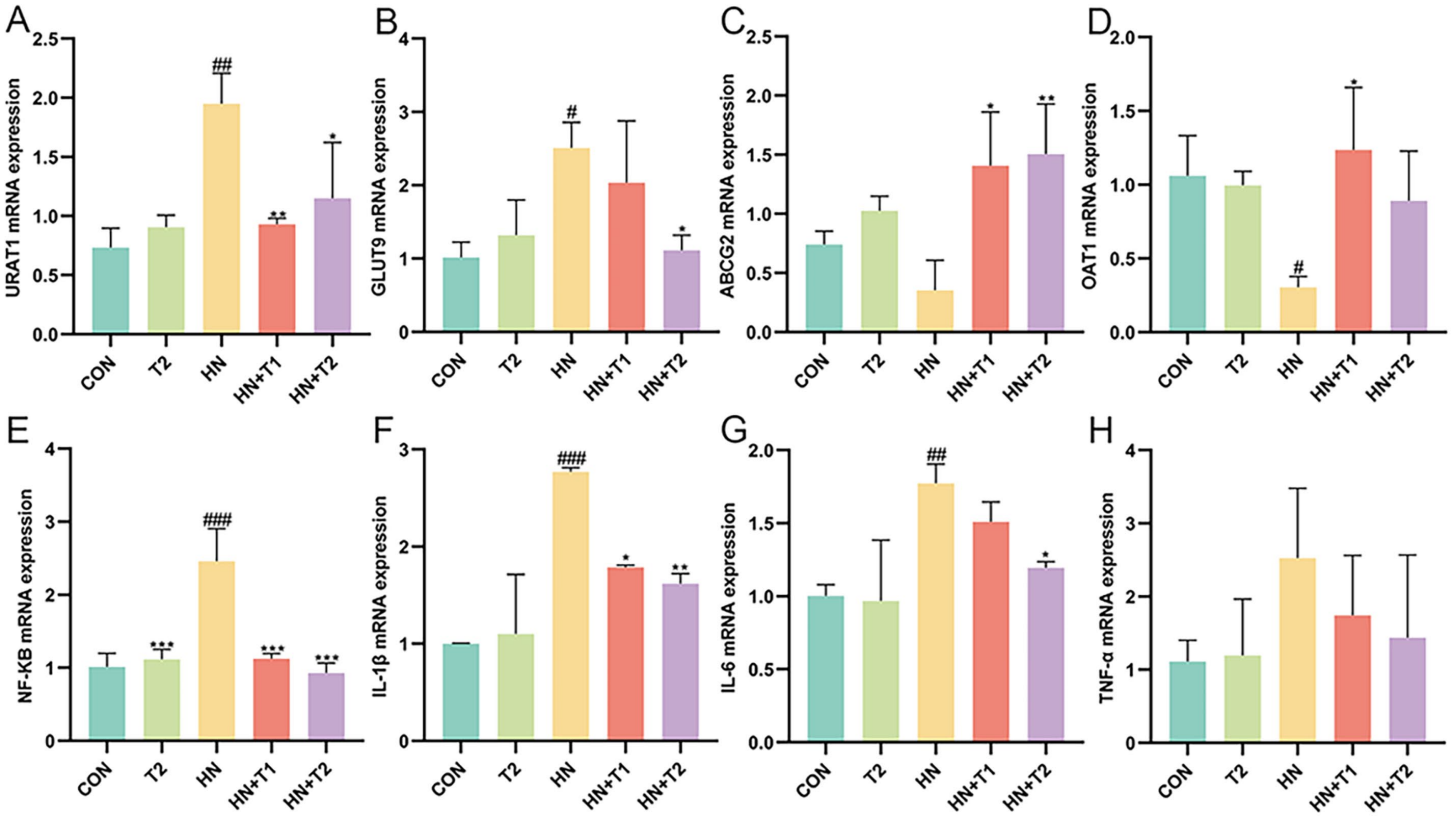
Effect of T on UA transporter and UA induced inflammation in HN rats. **(A−H)** The mRNA expression levels of URAT1, GLUT9, ABCG2, OAT1, NF-κB, IL-1β, IL-6, and TNF-α measured by qRT-PCR. Values were expressed in mean ± SEM (*n* = 6/group). ^#^*p* < 0.05, ^##^*p* < 0.01, ^###^*p* < 0.001 vs. CON, ^*^p < 0.05, ^**^p < 0.01, ^***^p < 0.001, vs. HN.

The excretion of UA through the kidneys relies on the particular mediation of UA reabsorption and excretion. Consequently, the expression of UA reabsorption and excretion transporters in the kidney was quantified through the use of qRT-PCR. As illustrated in Figures 2A−D, the expressions of UA reabsorption recombinant urate transporter 1 (URAT1) and glucose transporter 9 (GLUT9) were remarkably increased, while UA excretion adenosine triphosphate binding box transporter G2 (ABCG2) and organic anion transporter 1 (OAT1) showed significant decrease in HN rats (*p* < 0.05). The findings indicated that the reabsorption of UA was enhanced, whereas the excretion of UA was diminished in the kidneys of HN rats. In contrast to the HN group, the URAT1 and GLUT9 were inhibited by T at a dose of 1 and 2% in a dose-dependent manner. In addition, T was observed to significantly enhance the expression of the UA excretion transporter ABCG2 and OAT1 in the kidney (*p* < 0.05). The results yielded evidence that T may facilitate renal UA excretion by enhancing the expression of renal UA transporters in HN rats. Prior research has corroborated the existence of a correlation between HUA and renal inflammation, which can culminate in significant renal damage. Furthermore, the NF-κB signaling pathway may regulate the expression of UA transporters. Therefore, the impact of T on renal inflammation in rats with HN was evaluated. In comparison with the control group, the rats with HN displayed a notable elevation in the levels of NF-κB, IL-1β, IL-6, and TNF-α. Nevertheless, treatment with T at the specified concentrations markedly reduced elevated NF-κB, IL-1β, IL-6, and TNF-α in the kidney (Figures 2E−H, *p* < 0.05). The findings provided evidence that T may exert anti-inflammatory effects by modulating NF-κB activation in the kidneys of HN rats, which could potentially enhance renal function and facilitate UA excretion.

### 3.4 Taurine alleviated UA-induced oxidative stress and inflammation in HK-2 cells

**Figure 3:**
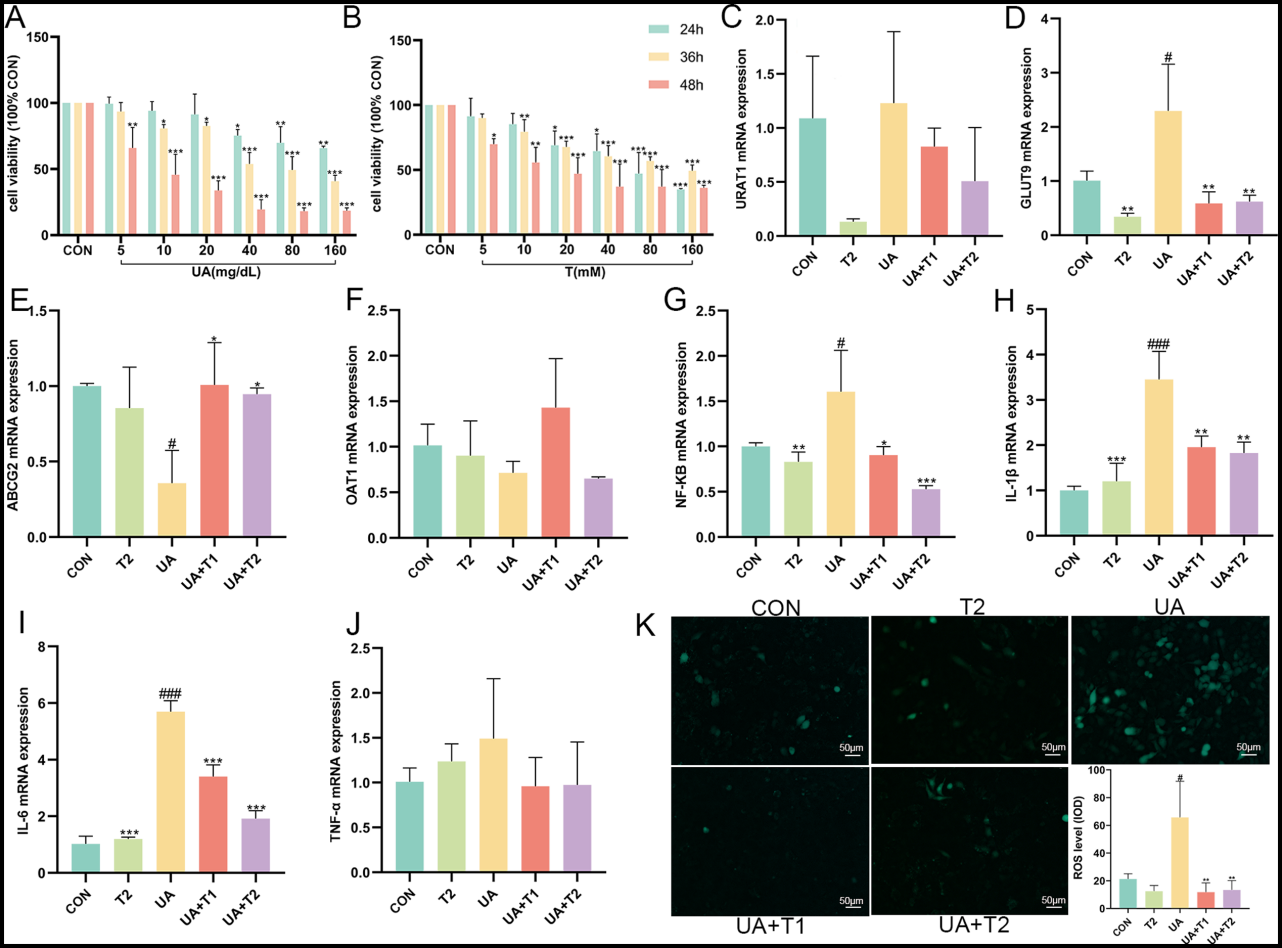
Effect of T on cell viability and UA induced inflammation and oxidative stress in HK-2 cells. **(A,B)** Cell viability. **(C−J)** The mRNA expression levels of URAT1, GLUT9, ABCG2, OAT1, NF-κB, IL-1β, IL-6, and TNF-α measured by qRT-PCR. **(K)** Changes of ROS. Values were expressed in mean ± SEM. ^#^*p* < 0.05, ^##^*p* < 0.01, ^###^*p* < 0.001 vs. CON, ^*^p < 0.05, ^**^p < 0.01, ^***^p < 0.001, vs. UA.

Prolonged exposure to UA causes tubular epithelial injury, which in turn drives the progression of interstitial fibrosis by activating autocrine and paracrine signals. Hence, the possible cytotoxicity impacts of UA (at concentrations of 5, 10, 20, 40, 80, and 160 mg/dL) and T (at concentrations of 5, 10, 20, 40, 80, and 160 mM) were determined using the CCK-8 assay (Figures 3A,B). As illustrated in Figure 3B, UA at concentrations not exceeding 40, 10, and 5 mg/dL at 24, 36, and 48 h showed no cytotoxicity on HK-2 cells. Notably, T significantly restored the cell viability in UA-induced HK-2 (*p* < 0.05). As shown in Figures 3C−F, the expression of URAT1 and GLUT9 was markedly elevated, whereas UA excretion via ABCG2 and OAT1 was remarkably decreased in UA-induced HK-2 cells (*p* < 0.05). These results suggested that the reabsorption of UA was enhanced, while the excretion of UA was diminished in UA-induced HK-2 cells. In contrast to the UA group, the activities of URAT1 and GLUT9 were restrained by T. In addition, T was observed to significantly enhance the expression of the UA excretion transporter ABCG2 and OAT1 in HK-2 cells (*p* < 0.05). The findings indicated that T might enhance renal UA excretion by enhancing the expression of renal UA transporters in HK-2 cells. Subsequently, the impact of T on the inflammatory response in UA-induced HK-2 cells was evaluated. In Figures 3G−K, it demonstrated that the levels of NF-κB, IL-1β, IL-6, TNF-α, and ROS were elevated in UA-stimulated HK-2 cells. In comparison with the control group, the administration of T led to a notable reduction in the production of several key inflammatory mediators, including NF-κB, IL-1β, IL-6, TNF-α, and ROS (*p* < 0.05). These results provided evidence that T may attenuate oxidative stress and inflammation in UA-induced HK-2 cells.

### 3.5 Taurine modified the structure of gut microbiota in HN rats

**Figure 4:**
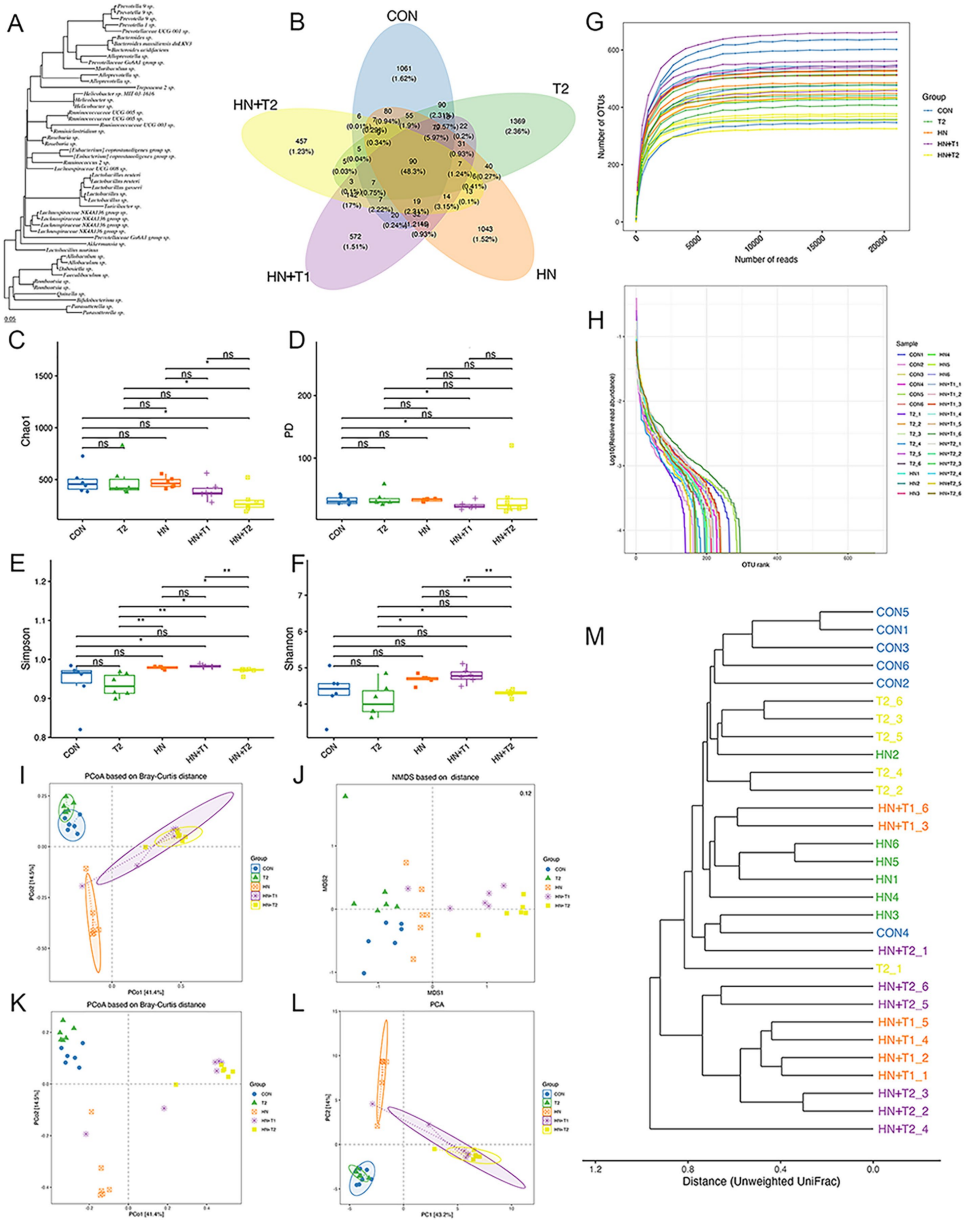
Effects of T on gut microbiota homeostasis in HN rats. **(A)** Phylogenetic tree of high abundance OUT. **(B)** Venn diagram analysis of differences in OTU distribution in each group. **(C)** The Chao1, **(D)** PD, **(E)** Simpson, and **(F)** Shannon index of each group. **(G)** The rarefaction curve of each sample. **(H)** OTU rank abundance curves. **(I)** PCoA based on Bray–Curtis distance with ellipses. **(J)** NMDS based on Jaccard distance. **(K)** PCoA based on Bray–Curtis distance. **(L)** The OUT-based PCA. **(M)** Clustering dendrogram based on unweighted UniFrac distance. Confidence ellipses represented the 95% confidence interval of each group. Values were expressed in mean ± SEM (*n* = 6/group). ^*^p < 0.05 and ^**^p < 0.01.

An increasing body of evidence indicates that gut dysbiosis is a pivotal factor in the pathogenesis of HN. Consequently, we conducted a 16S rRNA analysis to assess the potential impact of T on the intestinal flora in HN rats. In light of the increasing understanding of the impact of gut microorganisms on human health, the gut microbiota has been ascertained as a potential new target for HN therapy. To study which species are shared among different groups and which are unique, a Venn diagram is employed for the purpose of community analysis. An OTU abundance table is employed in the construction of a Venn diagram, where the presence of operational taxonomic units (OTUs) in each sample is statistically counted for each set. The OTUs are unique to each sample and the ones that are shared among different samples. Venn diagrams (Figures 4A,B) are a useful visual representation of the similarity of OTU composition. The portion of the petal that is superimposed represents the OTUs that are shared by adjacent groups. Each color corresponds to a distinct group. A total of 4,502 OTUs were generated from the five groups, with 1,061 unique OTUs observed in the CON group, 1,043 in the HN group, 1,369 in the T2 group, 457 in the HN + T2 group, and 572 in the HN + T1 group. These five groups shared 90 OTUs.

α-diversity is defined as the diversity of species within a specific region or ecosystem. The most commonly used α-diversity indices are Chao1, PD, Simpson, and Shannon. The main information includes the mean or median of the data and its dispersion. As shown in Figures 4C−H, the Chao1, PD, Simpson, and Shannon indexes remained largely unchanged in the HN group relative to the CON group. To the contrary, following gavage T treatment, the HN + T2 exhibited reduced community richness and diversity relative to the HN, and similarly, the observed decrease in Simpson values was statistically significant (*p* < 0.05). The detailed results of the α-diversity index are shown in Table 2.

**Table 2:** Summary of the α-diversity index for all samples.

Sample ID	Observed	Chao1	Shannon	Simpson	Coverage	PD
CON1	391	396.200	4.566	0.972	0.999	25.633
CON2	450	470.000	3.293	0.820	0.998	33.218
CON3	373	384.538	4.228	0.963	0.999	27.682
CON4	503	514.887	4.285	0.932	0.998	37.482
CON5	431	442.875	4.557	0.969	0.999	27.210
CON6	706	727.280	5.063	0.984	0.998	42.477
T2_1	792	829.513	4.842	0.968	0.997	58.521
T2_2	386	397.118	3.795	0.915	0.999	29.170
T2_3	368	379.875	4.196	0.948	0.999	25.571
T2_4	513	530.721	3.615	0.898	0.998	36.771
T2_5	411	419.775	3.791	0.913	0.999	30.051
T2_6	411	413.000	4.423	0.963	0.999	28.789
HN1	479	506.020	4.462	0.973	0.998	35.344
HN2	468	485.146	4.660	0.978	0.998	33.953
HN3	428	440.536	4.724	0.981	0.999	34.986
HN4	539	557.000	4.849	0.977	0.998	34.579
HN5	421	433.037	4.702	0.981	0.999	30.271
HN6	406	412.774	4.724	0.982	0.999	29.795
HN + T1_1	368	376.500	4.744	0.982	0.999	20.798
HN + T1_2	357	359.500	4.805	0.985	1.000	22.129
HN + T1_3	542	562.067	4.911	0.982	0.998	35.632
HN + T1_4	274	280.429	4.491	0.981	1.000	17.881
HN + T1_5	358	362.550	4.671	0.980	0.999	22.178
HN + T1_6	422	430.053	5.114	0.989	0.999	26.374
HN + T2_1	501	521.128	4.410	0.955	0.998	36.783
HN + T2_2	270	278.000	4.349	0.975	0.999	19.000
HN + T2_3	226	229.500	4.272	0.974	1.000	14.663
HN + T2_4	297	307.000	4.300	0.972	0.999	120.227
HN + T2_5	193	193.000	4.139	0.972	1.000	19.193
HN + T2_6	245	246.000	4.303	0.975	1.000	29.175

The composition of the microbial community of the CON group differed from that of the HN, T2, HN + T2, and HN + T1 groups, indicating that low and high doses of T intake influenced the overall composition of the intestinal flora. To conduct subsequent analyses based on various commonly used sample-to-sample distance metrics and to facilitate the observation of the extent of differences and the patterns of variation in differences among samples, the PCoA is employed for exploration. The PCoA based on the Bray–Curtis plot demonstrated that the CON and HN groups could not be entirely separated, suggesting that the HN group was found to exert an influence on the architecture of the intestinal flora. To reflect the differences among samples as a whole on a two-dimensional coordinate system, NMDS is selected for calculation. Its characteristic is to reflect samples in a multi-dimensional space in the form of points based on the information within the samples. The extent of discrepancy between samples is represented by the distance between points on the graph, ultimately obtaining a two-dimensional spatial positioning map of the samples. The smaller the stress value of the result, the better, when it is less than 0.2, this suggests that the NMDS analysis is a reliable method of data collection (Figures 4I−L). As illustrated in Figure 4M, the proximity of the samples within each group suggests a high degree of similarity in the community structure.

### 3.6 Taurine modified the composition of gut microbiota in HN rats

**Figure 5:**
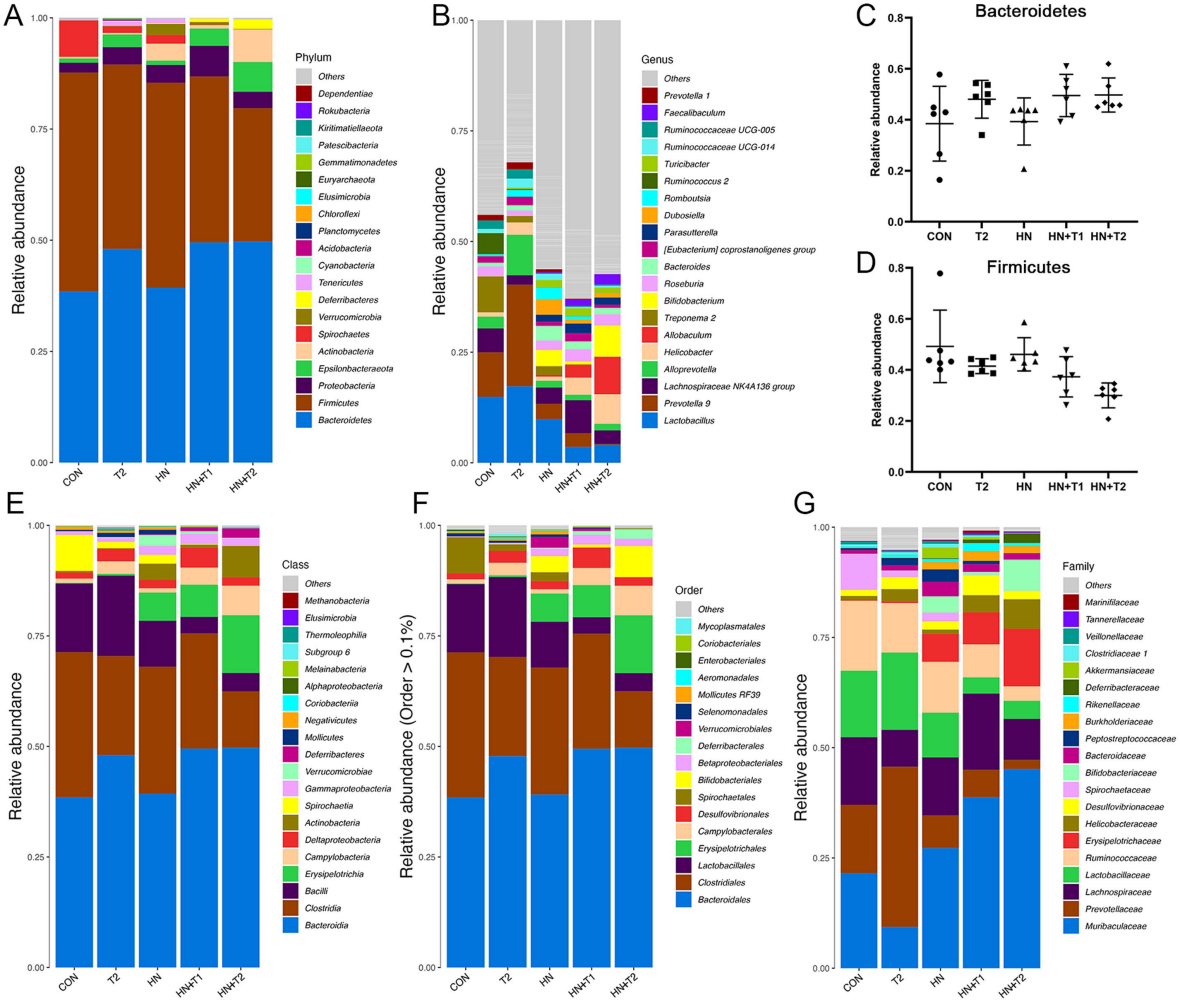
Effects of T-altered gut microbiota in HN rats. **(A)** Barplot of top 10 relative abundance at the phylum level. **(B)** Barplot of top 10 relative abundance at the genus level. **(C,D)** The relative abundance of Bacteroidetes and Firmicutes in each group, and the data were subjected by one-way ANOVA. Values were expressed in mean ± SEM (*n* = 6/group). **(E−G)** Barplot of top 10 relative abundance at class, order, and family level. ^*^p < 0.05, ^**^p < 0.01, and ^***^p < 0.001. Others represent the sum of the relative abundance of all species other than 10 and species without annotation information.

To present the data on species with high abundance visually, the 10 species with the highest abundance at the phylum, class, order, family, and genus taxonomic levels are selected for the generation of cumulative bar charts. A total of two bacterial species were identified at the phylum level within the intestinal flora. Subsequent sequence analysis of the CON, HN, T2, HN + T2, and HN + T1 samples revealed the presence of 27, 24, 30, 17, and 19 phyla, respectively. The phylum-level analysis (Figure 5A) revealed certain discrepancies in the composition of the intestinal flora between the groups. In conclusion, a total of 90% of the intestinal flora were identified as consisting primarily of the following three bacterial phyla: *Firmicutes*, *Proteobacteria,* and *Bacteroidetes*. The relative abundances of *Firmicutes* in CON, HN, T2, HN + T2, and HN + T1 were 49.2, 46.1, 41.5, 30.0, and 37.3%, separately. The relative abundances of *Bacteroidetes* in CON, HN, T2, HN + T2, and HN + T1 were 38.5, 39.3, 48.0, 49.7, and 49.5%, separately. As illustrated in Figure 5D, the proportion of *Firmicutes* in the HN was less than that observed in the CON. Concurrently, as can be observed from the data presented in Figure 5C, the relative abundance of *Bacteroidetes* in the HN was found to be lower than that observed in T2, HN + T2, and HN + T1, but it increased after T administration. A total of 293 genera were identified at the genus level across the five groups, and 258, 260, 255, 159, and 189 genera were obtained from CON, HN, T2, HN + T2, and HN + T1, respectively. The findings indicate that the predominant bacterial species within the intestinal flora of the five groups were *Lactobacillus*, *Lachnospiraceae NK4A136 group,* and *Prevotella 9* (Figure 5B). The relative abundances of *Lactobacillus* in CON, HN, T2, HN + T2, and HN + T1 were 14.9, 9.86, 17.3, 4.07, and 3.59%, separately. The relative abundances of *Lachnospiraceae NK4A136 group* in CON, HN, T2, HN + T2, and HN + T1 were 5.40, 3.71, 2.15, 3.07, and 7.49%, separately. The cumulative histogram of the top 10 most prevalent species generated at the taxonomic level of relative abundance for class, order, and family is displayed in Figures 5E−G.

**Figure 6:**
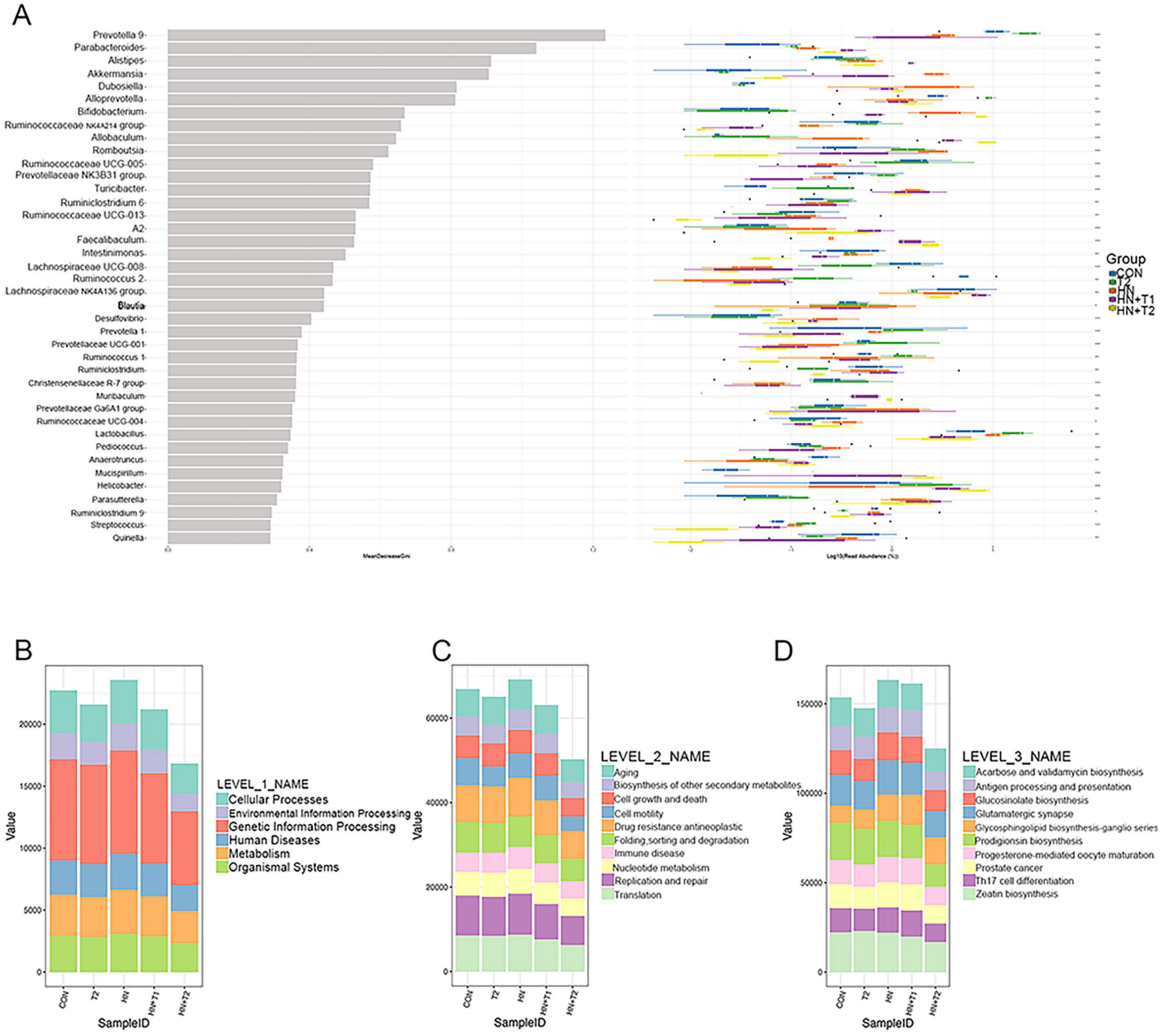
The result of random forest analysis and functional enrichment of the KO metabolic pathway at three different levels. **(A)** Random forest analysis. **(B)** KO analysis of level 1. **(C)** KO analysis of level 2. **(D)** KO analysis of level 3. The abscissa on the left is the mean decrease of the Gini index, the ordinate is the taxonomic information of the genus, and the box plot of the abundance of taxa in the right is the box plot of the abundance of different groups (Kruskal–Wallis rank-sum test). ^*^p < 0.05, ^**^p < 0.01, and ^***^p < 0.001.

The combination of random forest analysis and different tests allows for the identification of species exhibiting remarkable differences between groups. Furthermore, the method can be employed to identify crucial biomarkers. A total of 40 species exhibited remarkable discrepancy (Figure 6A). The potential metabolic function of the intestinal microbiota is attributed to alterations in the bacterial microbiome as documented in the KEGG database. The functional prediction outcomes can be further enriched at three levels of the metabolic pathways, as illustrated in Figures 6B−D. At the first level of each group, the abundance of cellular processes was the highest, while the abundance of organismal systems was the lowest. At the second level of each group, the abundance of translation is the lowest, followed by replication and repair and nucleotide metabolism. The metabolic profiles of immune-related diseases, folding, sorting and degradation, drug resistance, cell motility, and cell growth and death are analogous. The abundance of zeatin biosynthesis was found to be the lowest among the third level of each group, followed by Th17 cell differentiation. Prodigiosin biosynthesis and glycosphingolipid biosynthesis-ganglio series in diverse environments have little discrepancy in abundance. In reverse, glutamatergic synapse, glucosinolate biosynthesis, and acarbose and validamycin biosynthesis have little discrepancy in abundance. The results indicated that compared with CON, HN, and T2, the levels of metabolism, at both Level 1 and Level 2, as well as the metabolic pathways at Level 3, demonstrate a notable increase in the HN + T1 and HN + T2. It has been postulated that T can improve HN by augmenting the metabolic pathways related to metabolic regulation.

### 3.7 Taurine regulated metabolites in HN rats

**Figure 7:**
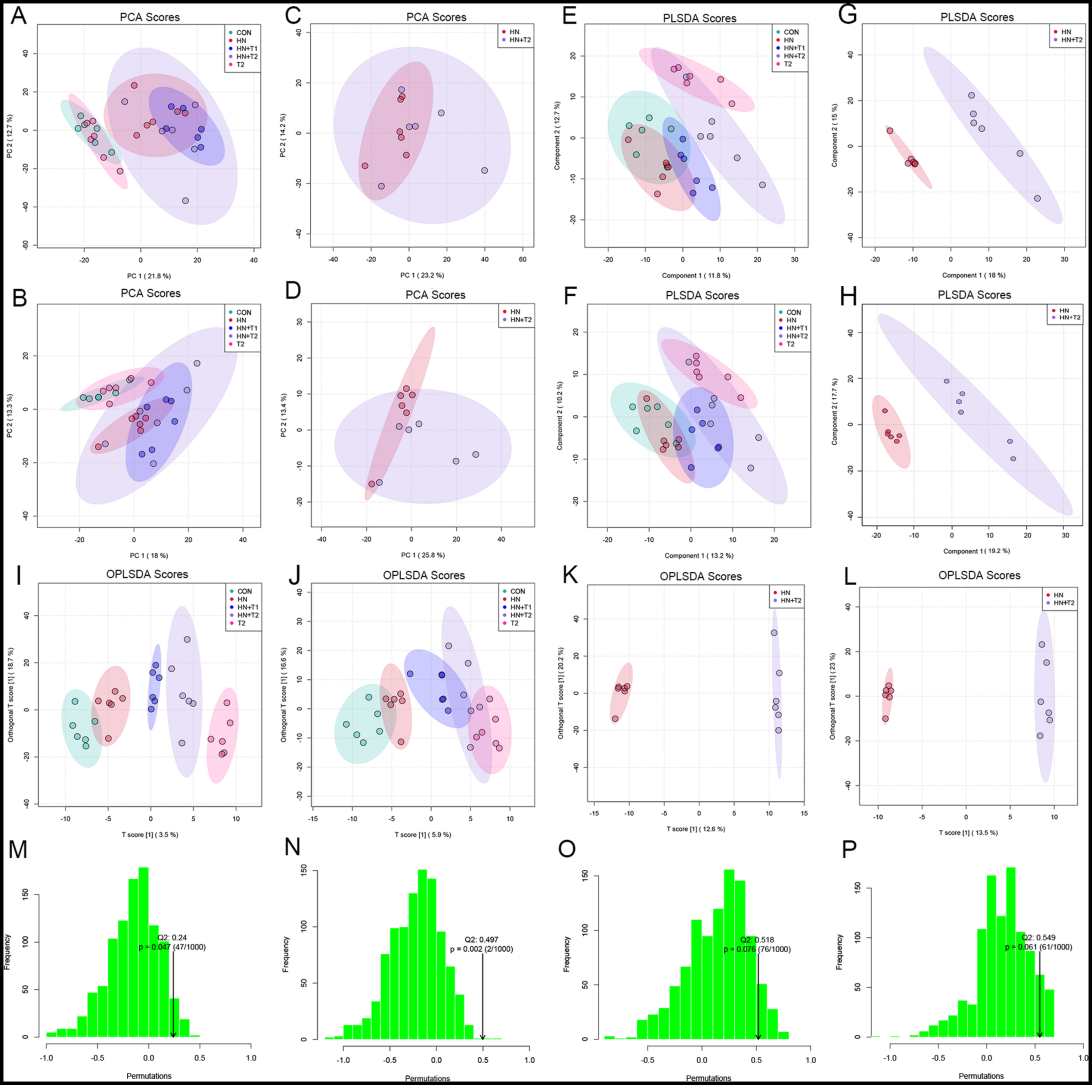
Effects of T on fecal metabolism. **(A)** Positive-ion mode PCA score plot for all groups. **(B)** Negative-ion mode PCA score plot for all groups. **(C)** Positive-ion mode PCA score plot for HN and HN + T2 groups. **(D)** Negative-ion mode PCA score plot for HN and HN + T2 groups. **(E)** Positive-ion mode PLS-DA score plot for all groups. **(F)** Negative-ion mode PLS-DA score plot for all groups. **(G)** Positive-ion mode PLS-DA score plot for HN and HN + T2 groups. **(H)** Negative-ion mode PLS-DA score plot for HN and HN + T2 groups. **(I)** Positive-ion mode OPLS-DA score plot for all groups. **(J)** Negative-ion mode OPLS-DA score plot for all groups. **(K)** Positive-ion mode OPLS-DA score plot for HN and HN + T2 groups. **(L)** Negative-ion mode OPLS-DA score plot for HN and HN + T2 groups. **(M)** Positive-ion mode PLS-DA 200-permutation test score plot. **(N)** Positive-ion mode OPLS-DA 200-permutation test score plot. **(O)** Negative-ion mode PLS-DA 200-permutation test score plot. **(P)** Negative-ion mode OPLS-DA 200-permutation test score plot. ^*^p < 0.05, ^**^p < 0.01 vs. HN.

PCA was performed for each group in both positive and negative ion mode (Figures 7A,B). Positive and negative ion patterns from PCA showed separation between HN and HN + T2 samples (Figures 7C,D). PLS-DA analysis (Figures 7E,F) and OPLS-DA analysis (Figures 7I,J) were conducted in both positive and negative ion modes across all groups. Positive and negative ion patterns from PLS-DA analysis (Figures 7G,H) and OPLS-DA analysis (Figures 7K,L) showed significant separation between HN and HN + T2 samples. Supervised models PLS-DA and OPLS-DA (*R*^2^Y = 0.998, *Q*^2^ = 0.518) and the results of 200 interaction validations provided further confirmation of the existence of significant differences in metabolic processes between the two states (Figures 7M,O). In the negative ion model, the isolation of the two states in the PCA plot, the capital mass parameters of the PLS-DA and OPLS-DA models (*R*^2^Y = 0.998, *Q*^2^ = 0.549) indicated that the two states were efficiently distinguished. The results were validated by 200 interaction verification analyses (Figures 7N,P).

**Figure 8:**
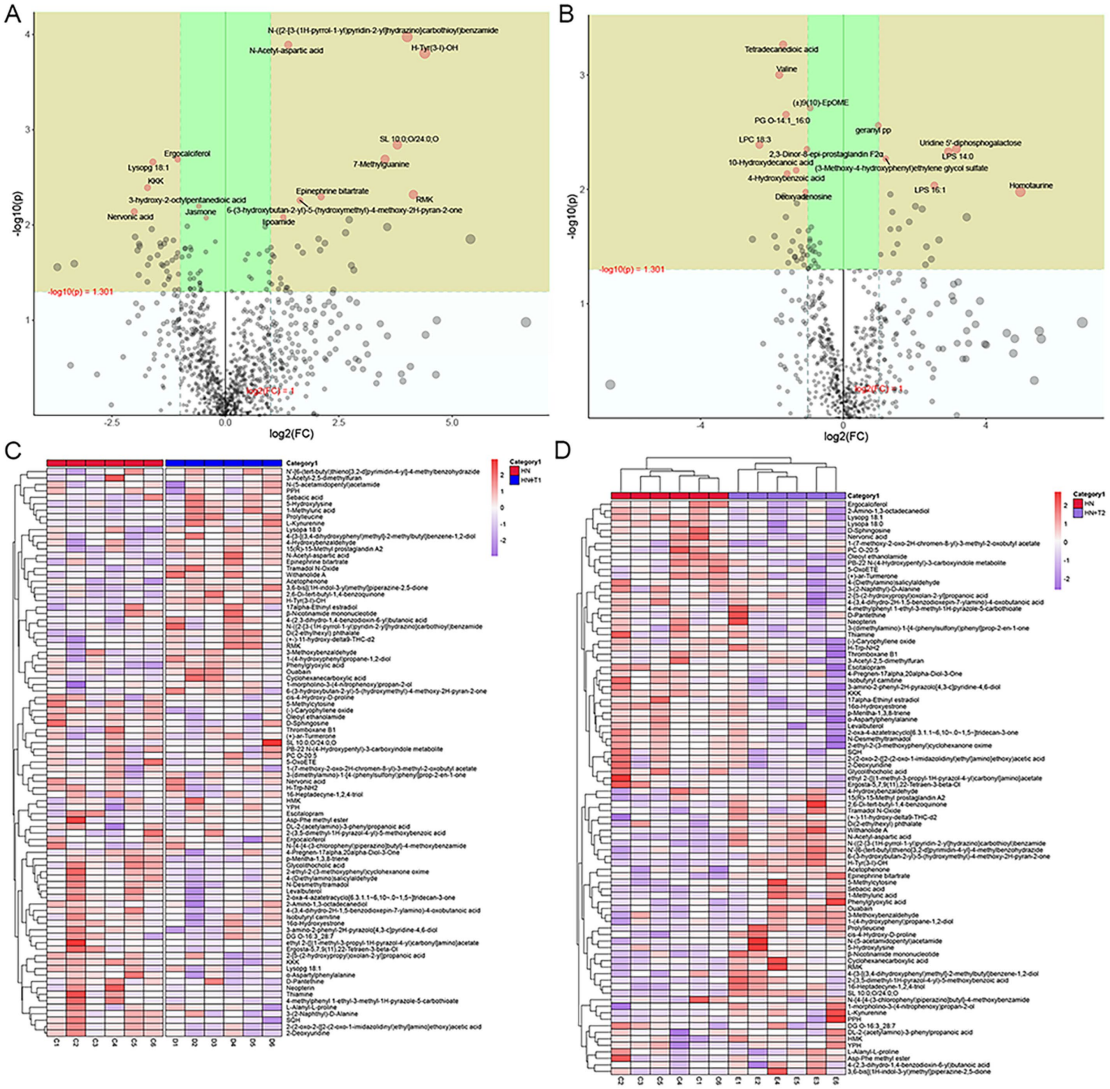
Effects of T on metabolites in HN rats. **(A)** Volcanic plot in positive-ion patterns. **(B)** Volcanic plot in negative-ion patterns. **(C)** Correlated heat maps of differential metabolites. **(D)** Heat map showing metabolite hierarchical clustering of significant differences in the positive-ion patterns.

Changes in the expression of different metabolites in the two ion patterns are shown in volcanic plot of multiplicative changes (Figures 8A,B). Correlation heat maps of the differential metabolites depict different patterns of metabolite changes between HN and HN + T1 (Figure 8C). Heat map of metabolite hierarchical clustering of significant differences in positive-ion patterns (Figure 8D).

### 3.8 Taurine regulated metabolic pathways in HN rats

**Figure 9:**
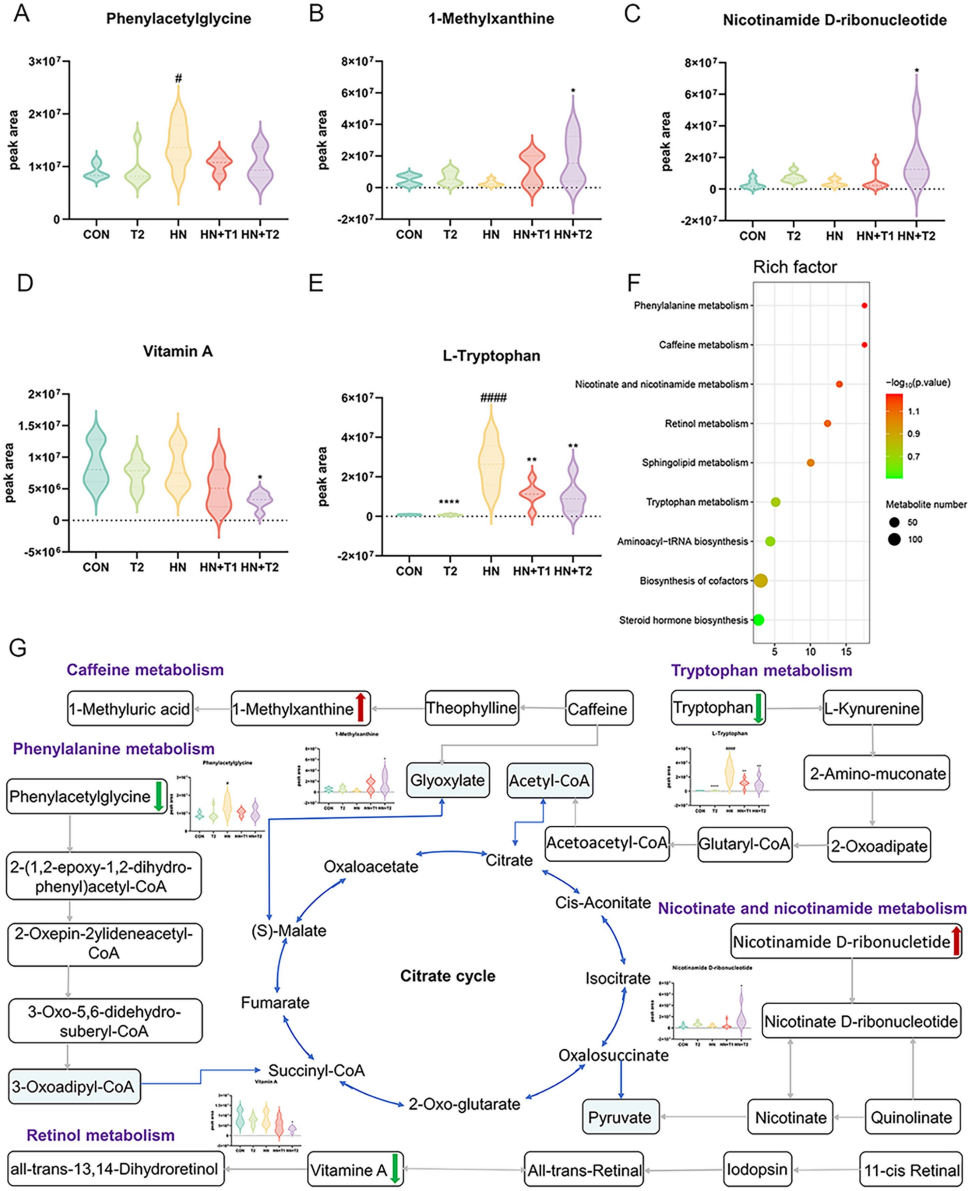
Effects of T-altered metabolic pathways in HN rats. **(A−E)** Differential metabolites for screening. **(F)** Bubble charts of metabolic pathway enrichment analysis. **(G)** Network schematic diagram of metabolites and involved pathways. (Red and green denote differential metabolites with significantly increased and decreased contents in the feces of HN + T2 rats).

KEGG enrichment analysis showed the top nine enriched pathways. Phenylalanine metabolism, caffeine metabolism, nicotinate and nicotinamide metabolism, retinol metabolism, sphingolipid metabolism, tryptophan metabolism, aminoacyl-tRNA biosynthesis, biosynthesis of cofactors, and steroid hormone biosynthesis pathways were enriched. KEGG pathway enrichment analysis is conducted through the application of Fisher’s exact test to calculate and analyze the significance of metabolite enrichment within the context of the metabolic pathway. A lower *p*-value suggests a greater degree of significance in the observed difference in the metabolic pathway. The outcome of the hierarchical clustering analysis of the significantly distinct metabolites between HN and HN + T2 groups is shown in Figure 9F. Metabolites clustered in similar clusters have similar expression patterns and may have comparable functions or share the same cellular pathways or metabolic processes. The dose–effect ties of the five differentiated metabolites are displayed in Figures 9A−E, including phenylacetylglycine, 1-methylxanthine, nicotinamide D-ribonucleotide, vitamin A, and L-tryptophan. Through the annotation and enrichment of metabolites between HN and HN + T2, before annotation and analysis of the KEGG pathway, 26 differential metabolites were obtained by merging the differentiated metabolites screened by positive and negative ion mode, and these are presented in Table 3, indicating that T may play a hypouricemic role through phenylalanine metabolism, caffeine metabolism, nicotinate, and nicotinamide metabolism, and other pathways. Notably, the majority of the metabolic pathways were found to be related to gut microbial metabolism, suggesting that the gut microbiota of HN rats is microbiologically dysregulated. Furthermore, pathway-related metabolic networks indicated that dissonance of metabolic pathways was also associated with the metabolism of purines, glycerophospholipid, alanine, arginine and proline, aspartate, and glutamate as well as the biosynthesis of steroid hormone, arginine. In summary, HN is closely associated with alterations in linoleic acid, glutamine, histidine, and pyrimidine metabolism.

**Table 3:** Differential metabolites of HN and HN + T2.

Number	Differential metabolites	*p*-value	m/z	RT [min]	Type
1	Ergocalciferol	0.001059	397.34602	10.16	down
2	Jasmone	0.005107	165.12777	5.469	down
3	Nervonic acid	0.005498	367.35674	10.742	down
4	7-Methylguanine	0.007154	166.0726	2.005	up
5	Phytosphingosine	0.009106	318.30014	7.417	down
6	Lipoamide	0.011986	206.06619	1.385	up
7	Ergosterol	0.012401	397.34693	10.729	down
8	Ouabain	0.014968	623.24689	6.233	up
9	Sebacic acid	0.016183	203.12825	2.271	up
10	Vitamin A	0.017766	269.22655	9.82	down
11	Mevastatin	0.019401	391.24604	8.503	down
12	1-Methylxanthine	0.019483	167.05679	4.807	up
13	L-Tryptophan	0.025967	409.1821	4.885	down
14	Glycerol-3-phosphate	0.025982	173.02125	1.546	down
15	Oxymatrine	0.026591	265.19159	5.464	down
16	Glu-Glu	0.029880	277.10345	1.386	up
17	Nicotinamide D-ribonucleotide	0.031117	335.06436	1.388	up
18	Cafestol	0.031449	317.21166	5.799	down
19	16 alpha-Hydroxyestrone	0.033288	287.16184	7.654	down
20	Cannabidiolic acid	0.034367	359.2204	6.652	down
21	Pepstatin	0.037875	686.4696	9.667	down
22	Isoferulic acid	0.041690	195.06569	5.517	up
23	Glycolithocholic acid	0.041899	434.32593	6.448	down
24	Diosgenin	0.042169	415.32105	9.992	down
25	Phenylacetylglycine	0.044836	211.10817	5.053	down
26	11-Ketoetiocholanolone	0.046696	305.21141	6.052	down

In parallel, we performed an interaction network of differentially expressed metabolites and associated metabolic pathways (Figure 9G). To better appreciate the potential pathogenesis of inflammation, we grouped differential metabolites into the following main categories associated with their biochemical functions: phenylalanine metabolism, caffeine metabolism, nicotinate and nicotinamide metabolism, retinol metabolism, and tryptophan metabolism.

### 3.9 Correlation analysis between microbiome and metabolomics

**Figure 10:**
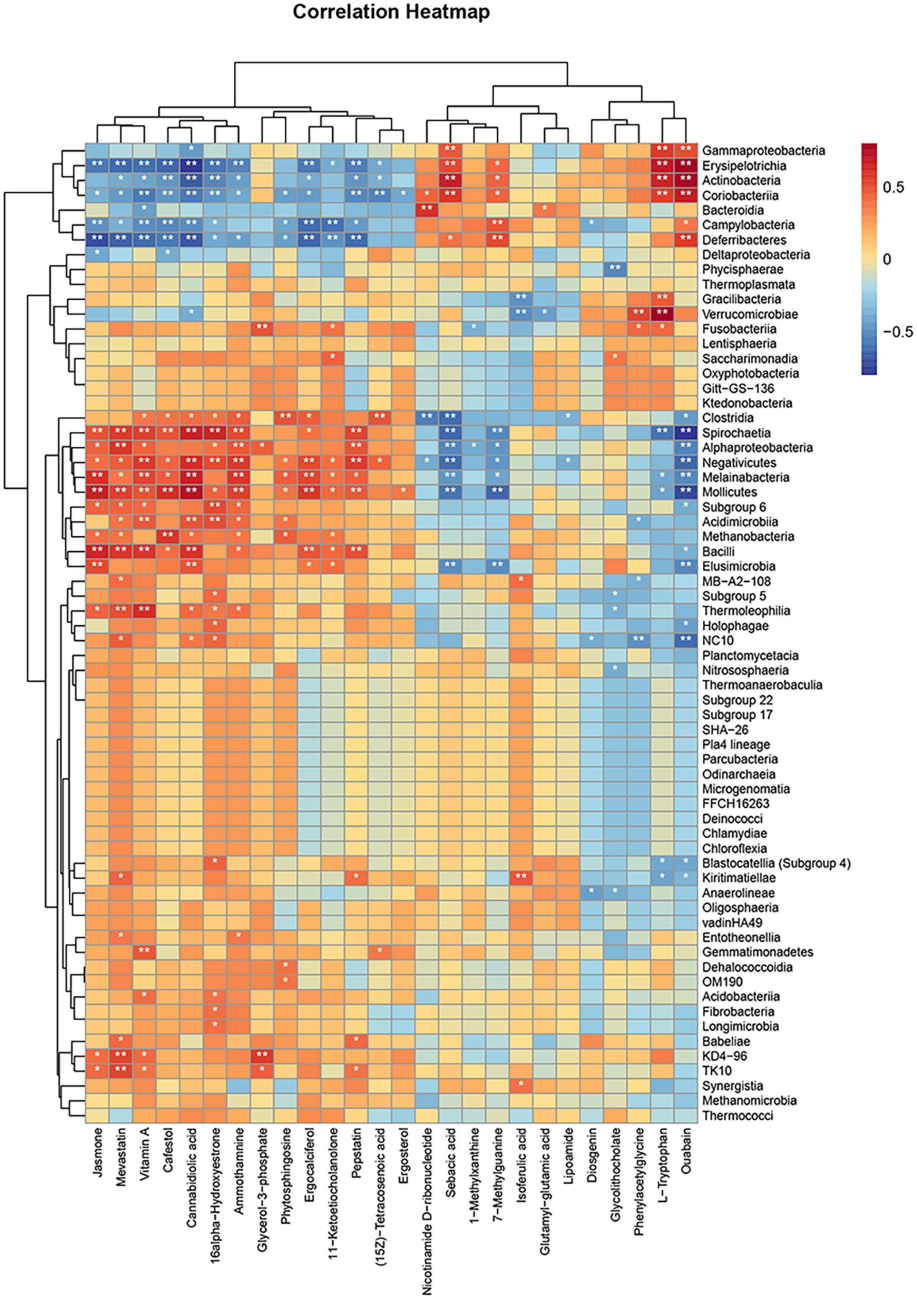
Hierarchical clustering heat map of Spearman’s correlation analysis of significantly different bacterial groups with significantly different metabolites. ^*^p < 0.05, ^**^p < 0.01, ^***^p < 0.001, vs. HN.

The purpose of this study was to analyze the relevance between microbiome and metabolomics, looking for probable variations in bacterial and metabolite populations with the potential to reveal relationships between bacterial composition and complex metabolites. A Spearman correlation analysis was employed to generate a correlation heatmap, with the objective of investigating the correlation between fecal metabolites and phyla (Figure 10). Similarities and discrepancy in the expression patterns of apparently different groups of bacteria and different metabolites were explored by performing Spearman’s correlation hierarchical clustering analysis on strain groups and metabolites. The analyses were conducted with the use of the R version 3.6.3 heatmap package. A grand total of 269 significant microbiota-fecal metabolite relevance were identified based on | *r* | > 0 and *p* < 0.05; among them, 127 microbiota-fecal metabolite correlations were identified based on *p* < 0.01, and the correlation was more significant. Specifically, *Spirochaetia*, *Erysipelotrichia*, *Verrucomicrobia*, *Erysipelotrichia*, *Actinobacteria*, *Mollicutes*, *Deferribacteres*, *Coriobacteriia*, *Melainabacteria*, *Bacilli*, *Negativicutes,* and *NC10* were associated notably fecal metabolites. It is possible to conclude that all fecal metabolites are associated with at least one class.

### 3.10 Correlation analysis between microbiome, metabolomics, and serum biochemical renal function indicators

**Figure 11:**
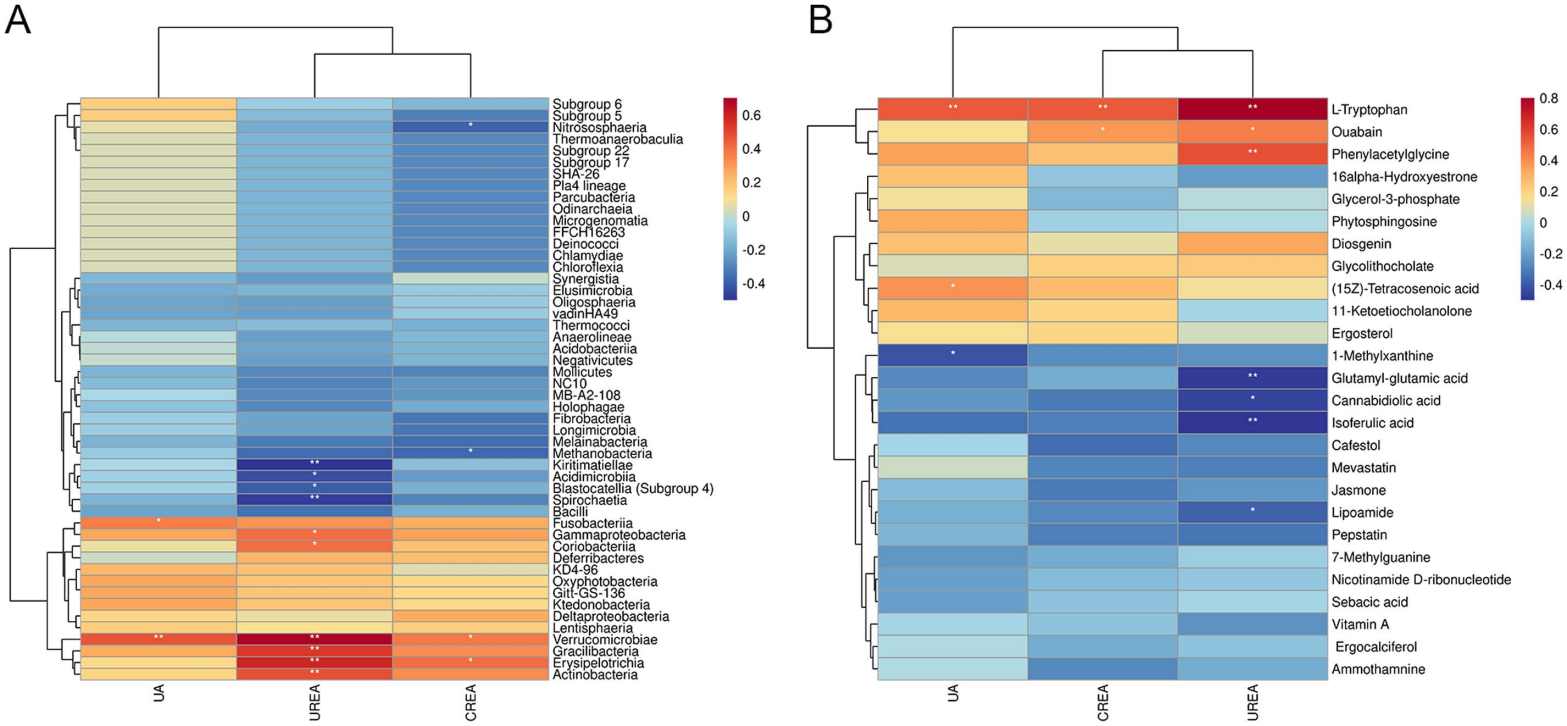
Correlation analysis between microbiome, metabolomics, and serum biochemical renal function indicators. **(A)** Hierarchical clustering heat map of Spearman’s correlation analysis of significantly different bacterial groups and serum biochemical renal function indicators (UA, UREA, and CREA). **(B)** Hierarchical clustering heat map of Spearman’s correlation analysis of differentially abundant metabolites and serum biochemical renal function indicators (UA, UREA, and CREA). ^*^p < 0.05, ^**^p < 0.01, ^***^p < 0.001, vs. HN.

To identify the relationships among the microbiome, metabolomics, and serum biochemical renal function indicators, Spearman’s correlation analysis was performed. As illustrated in Figure 11A, after T intervention, *Verrucomicrobia*, *Erysipelotrichia, Gracilibacteria*, *Actinobacteria*, *Coriobacteriia*, *Gammaproteobacteria*, and *Fusobacteriia* showed strong positive correlations with UA, UREA, and CREA, whereas *Kiritimatiellae*, *Spirochaetia*, *Acidimicrobiia*, *Blastocatellia (Subgroup 4)*, *Nitrososphaeria*, and *Methanobacteria* exhibited significant negative correlations with UA, UREA, and CREA (*p* < 0.05). In Figure 11B, the levels of UA, UREA, and CREA were significantly negatively correlated with glutamyl-glutamic acid, cannabidiolic acid, isoferulic acid, 1-methylxanthine, and lipoamide but showed significantly positive correlations with L-tryptophan, phenylacetylglycine, (15Z)-tetracosenoic acid, and ouabain (*p* < 0.05). Among these metabolites, L-tryptophan, phenylacetylglycine, and ouabain exhibited strong correlations with key renal function biomarkers (UA, UREA, and CREA).

## 4 Discussion

Our study showed that T has a therapeutic effect on HN rats, with the primary mechanisms of action being anti-inflammatory, and adjusting gut microbiota endogenous metabolites. Our evidence elucidating the complex pathogenesis of kidney disease from an intestinal microbial perspective opens up the possibility of developing innovative therapies in a large number of microbial pathways, both as potential pharmacological targets and mediators of kidney disease. T improved the adenine combined with ethambutol-induced HN (13) and offered prospects for the development of functional foods or drugs.

First of all, a suspension of adenine and ethambutol hydrochloride can be used to mimic purine metabolism disorders. This increases UA levels and induces a HUA model (26). The underlying pathological mechanism of HN is the excessive production or excretion of UA, which results in the accumulation of UA crystals in the kidney and an ensuing inflammatory response that impairs renal function (27). The combination of adenine with ethambutol hydrochloride has been the subject of several articles, in which it has been proposed as a model for the study of HUA-induced kidney damage (13, 28). The most commonly used indicators of kidney function are serum UREA and CREA levels (29). The results of animal experiments revealed that T ameliorated renal pathological changes in HN rats and reduced UA, CREA, and CRUA levels in rat serum. Therefore, these results suggest that T has an anti-HUA activity and may be able to protect against HUA-induced kidney damage.

Multiple processes are responsible for HN, with inflammatory injury and oxidative stress being the most significant contributors (30). It has been demonstrated in traditional research that deposition of UA crystals in renal tubules can result in active inflammosomes and induce inflammatory responses (31), while recent studies have concentrated on soluble UA, which has direct effects on localized inflammation and the generation of reactive oxygen species (32). For example, NF-κB is a pleiotropic transcription factor that regulates the expression of many genes involved in the inflammatory response during kidney injury and induces the release of proinflammatory cytokines such as TNF-α, IL-6, and IL-1β, which exacerbate kidney damage (33). UA induced the expression of TNF-α in vascular smooth muscle cells through the ROS-p38MAPK-NF-κB signaling pathway (34). In addition, ROS, a product of oxidative stress, has been related to the metabolism of urate and the production of ROS in high blood pressure and diabetes mellitus (35). In agreement with these findings, the HN group exhibited elevated levels of proinflammatory cytokines, whereas T decreased the levels of ROS IL-1β, IL-6, and TNF-α and inhibited the NF-κB pathway to reduce inflammation. Previous studies have shown that the mechanism of action involves the inhibition of urate transporter proteins and the promotion of high expression of urate excretion protein, and *Portulaca oleracea* L. is capable of reducing the reabsorption of urate by the body and thus facilitating its excretion (36). Fisetin was observed to exert a uricosuric effect through the modulation of renal urate transporter expression, including URAT1, OAT1, and ABCG2 (37). The same results were found in our study; in contrast to the HN group, T administration led to a significant reduction in URAT1 and GLUT9 levels, while T significantly enhanced UA excretion transporter ABCG2 and OAT1 expression in the kidney.

Dysbiosis of the gut microbiota is a pivotal factor in the etiology of HN (17), and some therapeutic microbial manipulations have been proven to be a promising therapeutic strategy (38, 39). Therefore, we investigated whether the gut microbiota contributes to its protective effect to reveal potential therapeutic mechanisms for T. The results demonstrate that T has a marked effect on the richness and diversity of the intestinal microflora, leading to the restoration of intestinal microecology in adenine- and ethambutol-induced HN rats. The assessment of α-diversity, β-diversity, and species composition substantiates this conclusion. A substantial body of evidence from scientific research indicates that the ratio of *Firmicutes* to *Bacteroidetes* is significantly correlated with the development of various metabolic disorders. Intriguingly, obese animal studies have confirmed the opposite trend (40). The findings indicated that the abundance of *Bacteroidetes* increased after T treatment and decreased for *Firmicutes*. Reportedly, *Lactobacillidae* have been reported to modulate pro-inflammatory pathways by decreasing cytokines such as IL-6 and TNF-α, inducing immune tolerance, and inhibiting T-cell effectors (41). *E. coli* in the human intestine produce XOD to influence UA production (42), and the *Lactobacillus* family inhibits UA accumulation indirectly by secreting rottlerin to inhibit the growth of *Escherichia coli* (43). Similarly, the *Lachnospiraceae NK4A136 group* has previously been linked to obesity and other associated metabolic disorders. The *Lachnospiraceae NK4A136 group* has been identified as a discriminatory feature of gut dysbiosis (44), and the results obtained may indicate a favorable effect of T on the regulation of HN; this reinforces the importance of the results of this study.

Aiming to achieve a comprehensive comprehension of the metabolic disorders caused by HUA, we investigated the impact of T on HN by metabolite profiling. In the HN + T2 vs. HN, we identified 26 significantly differential metabolites, and nine major metabolic pathways were obtained after the enrichment of KEGG, suggesting the potential effects of T on metabolic regulation. KEGG enrichment analysis showed the top 9 enriched pathways are as follows: phenylalanine metabolism, caffeine metabolism, nicotinate and nicotinamide metabolism, retinol metabolism, sphingolipid metabolism, tryptophan metabolism, aminoacyl-tRNA biosynthesis, biosynthesis of cofactors, and steroid hormone biosynthesis pathways were enriched. A reduction in the adherence of calcium oxalate crystals to caffeine-treated renal tubular epithelial cells has been demonstrated in a vitro study (45). Caffeine has the capacity to function as an adenosine inhibitor, inhibiting adenosine activity by competing to bind to its receptor (46). In addition, one study has reported a number of urine proteins that are altered after caffeine intake in association with an increase in urine volume. The proteomic data have demonstrated a reduction in urinary kininogen excretion, suggesting the potential for increased intrarenal kinin levels and, consequently, reduced vasopressin release, along with augmented renal vasodilation. The intrarenal vasodilatation thus increases the glomerular blood flow, the glomerular filtration rate, and the urine output (47). Nicotinamide is an inhibitor of 1 poly synthetase (PARS) activity. Nicotinamide adenine dinucleotide (NAD) acts as an electron carrier in the respiratory chain of the mitochondria, and the depletion of NAD results in a rapid fall in the intracellular level of ATP. In addition, nicotinamide can be recycled to NAD in a reaction that consumes ATP. Activation of PARS therefore leads to a decrease in ATP, which can ultimately lead to cell death (48–51). A study shows that urinary retinol is a specific sign of tubular damage in people with type 2 diabetes and that urinary retinol is a better indicator of proximal tubule dysfunction in people with type 2 diabetes compared to urinary retinol-binding protein or albumin (52). This suggests that T could improve HN by regulating phenylalanine metabolism, caffeine metabolism, nicotinate and nicotinamide metabolism, retinol metabolism, sphingolipid metabolism, and tryptophan metabolism.

A combination of 16S rRNA and metabolomics analyses was used to investigate the mechanisms of T regulation in HN model rats. The results showed a close relationship between several differentially expressed gut microbes and changes in fecal metabolites. We demonstrate that the gut microbiota and fecal metabolite profiles of HN rats differ significantly from those of normal animals and that the gut microbiota and fecal metabolites move further during the development of renal fibrosis. There is a strong correlation between metabolites and bacterial class, indicating that alterations in the gut microbiota are related to metabolic consequences.

Based on our comprehensive analysis, T has promising pharmacological effects on these targets, and then, we verified the discrepancy in the expression of these genes by qRT-PCR. This also supported our previous prediction. However, further internal mechanisms of interaction have not been explored, which is the direction of our future research.

## 5 Conclusion

In conclusion, our study sheds light on which T exerts its HN-improving effects through a variety of pathways, such as improving inflammation and modulating the gut microbiota and metabolic balance. These results showed that gut microbiota including *Lactobacillus* and *Lachnospiraceae NK4A136 group* showed correlations with nephroprotective profiles of T. Differentially abundant metabolites in feces were associated primarily with phenylalanine metabolism, caffeine metabolism, nicotinate and nicotinamide metabolism, retinol metabolism, and tryptophan metabolism. T decreased the levels of ROS IL-1β, IL-6, and TNF-α and inhibited the NF-κB pathway to reduce inflammation. Our study emphasizes that the potential protective mechanism of T for HN is not only related to altered metabolic pathways and downregulation of inflammatory cytokines but also to the reciprocal regulation of microbiota structure and metabolism. Although the definitive role of the gut microbiota needs further investigation, this research provides a novel idea for the study of T in the field of renal disease.

## Data Availability

The raw data supporting the conclusions of this article will be made available by the authors, without undue reservation.

## Footnotes

^1^ https://bitbucket.org/biobakery/biobakery/wiki/Home

^2^ https://www.mzcloud.org/

^3^ https://www.genome.jp/kegg/pathway.html

^4^ https://hmdb.ca/metabolites

^5^ http://www.lipidmaps.org/

## Glossary

ABCG2: adenosine triphosphate binding box transporter G2
CCK-8: cell counting kit-8
CKD: chronic kidney disease
CREA: creatinine
GLUT9: glucose transporter 9
H&E: hematoxylin and eosin
HN: hyperuricemia-induced nephropathy
HUA: hyperuricemia
LEfSe: linear discriminant analysis effect size
Masson: Masson’s trichrome
NMDS: non-metric multidimensional scaling
NF-κB: nuclear factor kappa-B
OAT1: organic anion transporter 1
OPLA-DA: orthogonal partial least squares-discriminant analysis
PAS: periodic acid-Schiff
PCA: principal component analysis
PCoA: principal coordinate analysis
PLS-DA: partial least squares-discriminant analysis
PSA: periodic acid-Schiff
qRT-PCR: quantitative real-time polymerase chain reaction
ROS: reactive oxygen species
TNF-α: tumor necrosis factor-alpha
UA: uric acid
UPLC-MS/MS: high-performance liquid chromatography–electrospray tandem mass spectrometry
URAT1: recombinant urate transporter 1
UREA: urea nitrogen
VIP: variable importance in projection
XOD: xanthine oxidase
IL-1β: interleukin-1β
IL-6: interleukin-6
